# Phenotype-Specific Gradients of NT-proBNP Reflect Distinct Functional and Structural Remodeling Signatures in Heart Failure

**DOI:** 10.3390/jcm15134957

**Published:** 2026-06-25

**Authors:** Sameh A. Ahmed, Osama M. Alhadramy, Lobna S. Hazman, Hussein M. Ismail

**Affiliations:** 1 Department of Pharmacognosy and Pharmaceutical Chemistry, College of Pharmacy, Taibah University, Al-Madinah Al-Munawarah 42353, Saudi Arabia; 2 Department of Internal Medicine, College of Medicine, Taibah University, Al-Madinah Al-Munawarah 42353, Saudi Arabia; ohadramy@taibahu.edu.sa (O.M.A.); hismail@taibahu.edu.sa (H.M.I.); 3 Adult Cardiology, Madinah Cardiac Center, Al-Madinah Al-Munawarah 42351, Saudi Arabia; lhazman@moh.gov.sa; 4 Department of Cardiology, College of Medicine, Suez Canal University, Ismailia 41522, Egypt

**Keywords:** NT-proBNP levels, heart failure phenotypes, cardiac remodeling, NYHA functional class, left ventricular ejection fraction

## Abstract

**Background/Objectives:** Heart failure (HF) classification based on left ventricular ejection fraction (LVEF) provides an incomplete representation of disease complexity, as it does not fully integrate functional impairment, structural remodeling, and clinical severity within a unified framework. Although N-terminal pro-B-type natriuretic peptide (NT-proBNP) is widely used for diagnosis and risk stratification, prior studies have primarily evaluated its role in isolation or within individual HF phenotypes, leaving its phenotype-specific distribution and integrative capacity across the HF spectrum insufficiently defined. This study aimed to address this gap by systematically evaluating NT-proBNP across HF phenotypes and assessing its potential as an integrative biomarker linking ventricular dysfunction, structural remodeling, and clinical severity. **Methods:** A cross-sectional study was conducted including 125 participants, comprising 65 clinically stable HF patients and 60 age- and sex-matched controls. HF patients were stratified according to LVEF into HF with reduced EF (HFrEF) (*n* = 28), (HFmrEF) (*n* = 20), and HF with preserved EF (HFpEF) (*n* = 17). Serum NT-proBNP concentrations were measured using a standardized electrochemiluminescence immunoassay. Clinical and echocardiographic parameters, including LVEF, left ventricular end diastolic diameter (LVEDD), left atrial diameter (LAD), and New York Heart Association (NYHA) functional class, were recorded and analyzed. **Results:** NT-proBNP levels were significantly higher in HF patients compared with controls (1845 ± 620 vs. 95.7 ± 40.5 pg/mL; *p* < 0.001) and demonstrated a clear stepwise increase across phenotypes (HFrEF: 2850.6 ± 710.4; HFmrEF: 1620.8 ± 480.2; HFpEF: 920.9 ± 310.3 pg/mL; *p* < 0.001). NT-proBNP showed a strong inverse correlation with LVEF (r = −0.68, *p* < 0.001) and significant positive correlations with LVEDD (r = 0.61, *p* < 0.001) and LAD (r = 0.57, *p* < 0.001). Higher levels were associated with more advanced NYHA functional class (III–IV vs. II: 2510 ± 680 vs. 980 ± 340 pg/mL; *p* < 0.001). ROC analysis demonstrated robust discriminatory performance across HF phenotypes, with the highest accuracy observed in HFrEF. **Conclusions:** NT-proBNP exhibits a phenotype-dependent gradient and consistently reflects ventricular dysfunction, adverse structural remodeling, and clinical severity across the HF spectrum. These findings support its role as an integrative biomarker that captures the multidimensional nature of HF, with potential implications for phenotype-based risk stratification and more precise clinical decision making.

## 1. Introduction

Heart failure (HF) represents one of the most pressing challenges in modern cardiovascular medicine, affecting more than 64 million individuals globally and accounting for substantial morbidity, mortality, and healthcare expenditure [1]. Despite remarkable advances in pharmacological therapies and device-based interventions over the past three decades, the prognosis of patients with HF remains disappointingly poor, with persistently high rates of hospitalization and premature death [2]. This enduring clinical challenge reflects a fundamental truth about HF: it is not a single disease entity, but rather a heterogeneous clinical syndrome that arises from diverse etiologies and unfolds along distinct pathophysiological trajectories [3].

The contemporary classification of HF, grounded in left ventricular ejection fraction (LVEF), has brought considerable clarity to clinical practice by defining three major phenotypes: heart failure with reduced ejection fraction (HFrEF), heart failure with mildly reduced ejection fraction (HFmrEF), and heart failure with preserved ejection fraction (HFpEF) [4,5]. This framework has proven valuable for guiding therapeutic decisions and structuring clinical trials, as each category differs meaningfully in underlying mechanisms, patterns of cardiac remodeling, and response to treatment [6]. Yet clinicians and researchers alike have increasingly recognized that LVEF alone, while pragmatic, offers an incomplete portrait of the disease. It does not, and cannot, fully capture the complex interplay between neurohormonal activation, ventricular remodeling, diastolic dysfunction, and the functional limitations that patients experience in their daily lives [7]. There is, therefore, a growing imperative to identify biomarkers that extend beyond echocardiographic classification and provide a more holistic assessment of disease burden.

N-terminal pro-B-type natriuretic peptide (NT-proBNP) has emerged as one of the most extensively validated biomarkers in cardiovascular medicine. Secreted primarily by ventricular cardiomyocytes in response to increased wall stress and volume overload, NT-proBNP is firmly embedded in international clinical guidelines for the diagnosis, risk stratification, and prognostic evaluation of HF [8,9,10]. Its measurement is widely accessible, analytically robust, and clinically familiar. However, the interpretation of NT-proBNP levels is not always straightforward. Circulating concentrations are influenced by a range of non-cardiac factors including advancing age, impaired renal function, obesity, and various metabolic comorbidities which may confound their interpretation in individual patients [11,12]. Beyond these practical considerations lies a more fundamental scientific question that remains incompletely answered: to what extent does NT-proBNP reflect not merely hemodynamic stress, but the broader landscape of cardiac remodeling and clinical deterioration that defines the HF syndrome.

Emerging evidence suggests that NT-proBNP may carry information about the structural underpinnings of HF, including left ventricular dilation and left atrial enlargement processes that are central to disease progression and strongly predictive of adverse outcomes [13]. Yet the existing literature reveals a fragmented picture. Studies conducted in HFrEF populations have demonstrated robust correlations between NT-proBNP and ventricular dimensions, but these findings cannot be assumed to generalize to HFpEF, where the pathophysiology and remodeling patterns are fundamentally different [14,15]. Investigations focusing on HFpEF, in turn, have linked NT-proBNP to diastolic dysfunction and filling pressures, yet have rarely included direct comparisons with HFrEF or HFmrEF groups within the same study [16]. Meanwhile, studies that have spanned multiple phenotypes often report mean biomarker levels across groups without systematically examining whether the strength of association between NT-proBNP and remodeling indices varies by phenotype [17,18]. The consequence of this fragmented evidence base is a persistent and clinically important knowledge gap: it remains unclear whether NT-proBNP functions consistently as an integrative biomarker, one that simultaneously reflects systolic dysfunction, adverse structural remodeling, and clinical symptom burden across the full HF spectrum. To the best of our knowledge, no prior study has addressed this question by combining comprehensive phenotypic stratification, multi-parameter echocardiographic phenotyping, and standardized clinical severity assessment within a single, well-characterized cohort. To the best of our knowledge, few studies have simultaneously evaluated HFrEF, HFmrEF, and HFpEF within a single cohort using a unified analytical framework that examines NT-proBNP in relation to ventricular function, structural remodeling, and symptom burden. Consequently, it remains uncertain whether the biomarker exhibits a consistent phenotype-dependent pattern across the full spectrum of HF severity.

This gap carries direct clinical relevance. In an era moving toward precision medicine and phenotype-guided management, clinicians need biomarkers that bridge the divide between what is measured on echocardiography and what patients experience symptomatically. A biomarker capable of reflecting the multidimensional nature of HF could refine risk stratification, guide therapeutic intensity, and potentially serve as a surrogate endpoint in clinical trials targeting reverse remodeling [19].

The present study was therefore designed to close this gap by addressing several interrelated aims within a unified analytical framework. The study sought to quantify and compare serum NT-proBNP concentrations across HFrEF, HFmrEF, HFpEF, and age- and sex-matched controls without HF, with the hypothesis that NT-proBNP levels would exhibit a phenotype-dependent gradient reflecting increasing disease severity. It further aimed to determine the strength, direction, and consistency of associations between NT-proBNP and key echocardiographic indices, including LVEF as a measure of systolic function, and both left ventricular end-diastolic diameter (LVEDD) and left atrial diameter (LAD) as measures of adverse structural remodeling, testing whether these associations persist across all phenotypes or are attenuated in specific subgroups. Finally, the study evaluated the relationship between NT-proBNP levels and clinical symptom burden, as assessed by New York Heart Association (NYHA) functional class, to determine whether the biomarker captures not only structural and functional derangements but also their symptomatic expression. Collectively, these analyses tested the central hypothesis that NT-proBNP serves not merely as a conventional diagnostic marker, but as a multidimensional indicator of the HF phenotype that reflects the full burden of disease.

## 2. Materials and Methods

### 2.1. Study Design and Setting

This investigation was designed as a single-center, cross-sectional observational study conducted at the Madinah Cardiac Center, Al-Madinah Al-Munawarah, Saudi Arabia, over a 12-month period from October 2024 to September 2025. The study protocol was developed in accordance with the Declaration of Helsinki and was approved by the Institutional Review Board of the Madinah Cardiac Center (Approval No.: H-03-M-143; approval date: 14 October 2024). Written informed consent was obtained from all participants prior to any study-related procedure. The cross-sectional design was selected to enable simultaneous assessment of biochemical, echocardiographic, and clinical parameters within a single cohort, permitting comprehensive between-group comparisons under standardized conditions.

### 2.2. Study Population

A total of 125 participants were consecutively recruited during the study period, comprising 65 patients with clinically stable chronic HF and 60 age- and sex-matched control subjects without clinical evidence of HF. HF was diagnosed according to current guideline criteria, requiring typical symptoms and/or signs together with echocardiographic evidence of cardiac structural or functional abnormality. Patients were stratified into three phenotypic groups based on LVEF measured at enrollment: HFrEF (LVEF < 40%), HFmrEF (LVEF 40–49%), and HFpEF (LVEF ≥ 50%). Eligibility criteria were defined a priori. Inclusion criteria for HF patients were: age ≥ 18 years, a confirmed diagnosis of chronic HF, and stable NYHA functional class for at least four weeks prior to enrollment, absence of acute decompensation on physical examination, HF-related hospitalization, or major changes in HF pharmacotherapy. Control subjects were recruited from among individuals undergoing routine clinical evaluation at the same center and were required to have no history or clinical evidence of HF and a preserved LVEF. The required sample size was estimated a priori using G*Power software (version 3.1.9.7; Heinrich-Heine Universität Düsseldorf, Düsseldorf, Germany). For a one-way ANOVA comparing four independent groups, assuming a medium to large effect size (Cohen’s f = 0.35), an alpha level of 0.05, and a statistical power of 80%, the minimum total sample size was calculated to be 112 participants. The final enrolled sample of 125 participants exceeded this threshold, providing adequate power for the planned between-group comparisons. Exclusion criteria applied to all participants included: acute coronary syndrome within the preceding three months; hemodynamically significant valvular heart disease (moderate or greater stenosis or regurgitation); congenital heart disease; severe renal impairment (estimated glomerular filtration rate < 30 mL/min/1.73 m^2^); active infection or inflammatory disease; known malignancy; and any condition judged likely to influence NT-proBNP concentrations independently of cardiac status. Individuals receiving investigational therapies or with incomplete clinical or echocardiographic data were also excluded. All HF patients were receiving guideline-directed medical therapy as clinically indicated, including renin-angiotensin system inhibitors, beta-blockers, mineralocorticoid receptor antagonists, and diuretics. Pharmacotherapy had been stable for at least four weeks prior to enrollment, reducing intra-patient variability in NT-proBNP levels attributable to recent treatment changes.

### 2.3. Clinical and Echocardiographic Assessments

Demographic data, medical history, and medication use were collected through structured patient interviews and verified against medical records. Comorbid conditions, including hypertension, diabetes mellitus, and dyslipidemia, were systematically recorded based on documented diagnoses and current treatment. Clinical assessment included general physical examination and determination of the NYHA functional class by a cardiologist blinded to NT-proBNP results. Comprehensive transthoracic echocardiography was performed using a Vivid E95 ultrasound system (GE Healthcare, Chicago, IL, USA). All measurements were acquired according to the recommendations of the American Society of Echocardiography and the European Association of Cardiovascular Imaging [20]. LVEF was calculated using the modified Simpson’s biplane method of disks from apical four-chamber and two-chamber views. LVEDD was measured from parasternal long-axis M-mode recordings at the level of the mitral valve leaflet tips. LAD was measured from the parasternal long-axis view at end systole. All echocardiographic measurements represented the average of three consecutive cardiac cycles in sinus rhythm or five cycles in atrial fibrillation. To minimize operator-related variability, studies were performed by two cardiologists whose inter-observer agreement had been established prior to study initiation (intraclass correlation coefficient >0.90 for all reported parameters).

### 2.4. Laboratory Measurements

Venous blood samples were collected from all participants following an overnight fast of at least eight hours. Samples were processed within 30 min of collection by centrifugation at 3000 rpm for 10 min, and serum was aliquoted and stored at −80 °C until analysis. Laboratory evaluations included high-sensitivity troponin I, lipid profile (low-density lipoprotein (LDL), high-density lipoprotein (HDL), triglycerides), and glycated hemoglobin (HbA1c), all measured using automated analyzers according to standard procedures. Serum NT-proBNP concentrations were quantified using an electrochemiluminescence immunoassay (ECLIA) on a Cobas e immunoassay analyzer (Roche Diagnostics, Basel, Switzerland) with the Elecsys proBNP II kit (Roche Diagnostics, Mannheim, Germany). The analytical measurement range of the assay was 5–3500 pg/mL. The intra-assay coefficient of variation was <3% and the inter-assay coefficient of variation was <5% across the range of concentrations observed in this study. All assays were performed in duplicate, and the mean value was used for analysis. Laboratory personnel performing NT-proBNP measurements were blinded to all clinical and echocardiographic data.

### 2.5. Statistical Analysis

Statistical analyses were performed using SPSS software (version 21.0; IBM Corp., Armonk, NY, USA). Data were examined for completeness, distributional properties, and outliers prior to hypothesis testing. Continuous variables are presented as mean ± standard deviation (SD), and categorical variables are expressed as frequencies and percentages. For comparisons across the four study groups (controls, HFpEF, HFmrEF, HFrEF), one-way analysis of variance (ANOVA) was applied, followed by Tukey’s honestly significant difference (HSD) post hoc test for pairwise comparisons when the overall F-test was significant. Between-group effect size was quantified using partial eta squared (η^2^) for the overall ANOVA and Cohen’s d for pairwise comparisons. Linear trend analysis (P-trend) was performed across groups ordered by increasing HF severity (controls → HFpEF → HFmrEF → HFrEF) using polynomial contrast analysis within the ANOVA framework. Relationships between NT-proBNP and continuous clinical, biochemical, and echocardiographic variables were assessed using Pearson’s correlation coefficient with corresponding 95% confidence intervals. Associations between NT-proBNP and NYHA functional class were examined using point-biserial correlation (NYHA III–IV vs. I–II) within each HF phenotype. To identify independent determinants of NT-proBNP, multivariable linear regression analysis was performed with log-transformed NT-proBNP as the dependent variable. Log transformation was applied to normalize the positively skewed distribution of NT-proBNP values. All variables that were clinically relevant or demonstrated associations in unadjusted analyses were entered simultaneously into the model. Results are reported as unstandardized regression coefficients (β) with standard errors, standardized coefficients, and 95% confidence intervals. Model assumptions were verified by inspection of residual plots (normality, homoscedasticity) and calculation of variance inflation factors (VIF); a VIF < 5 was considered indicative of acceptable multicollinearity. Model fit was assessed using R^2^ and adjusted R^2^. Diagnostic performance of NT-proBNP for discriminating HF phenotypes from controls was evaluated using receiver operating characteristic (ROC) curve analysis. The area under the ROC curve (AUC) with 95% confidence intervals was calculated for each HF phenotype. All statistical tests were two-tailed, and a *p*-value < 0.05 was considered statistically significant.

## 3. Results

### 3.1. Baseline Characteristics of Study Participants Stratified by HF Phenotypes

A total of 125 participants were enrolled, comprising 65 patients with HF and 60 age- and sex-matched controls. Baseline demographic and cardiovascular risk characteristics are summarized in Table 1. No statistically significant differences were observed across the four study groups in age, sex distribution, body mass index, or the prevalence of diabetes mellitus, hypertension, or smoking (all *p* > 0.05). These findings confirm comparability of the study groups with respect to major demographic variables and cardiovascular risk factors.

### 3.2. Clinical, Biochemical, and Echocardiographic Characteristics Across Study Groups

Clinical, biochemical, and echocardiographic parameters stratified by study group are presented in Table 2. LVEF demonstrated a progressive decline from controls (61.5 ± 3.8%) through HFpEF (56.8 ± 4.1%) and HFmrEF (44.6 ± 3.2%) to HFrEF (32.4 ± 5.1%; overall *p* < 0.001; P-trend < 0.001). Indices of cardiac remodeling followed a reciprocal pattern, with LVEDD and LA diameter increasing significantly across the same gradient (LVEDD: 48.2 ± 3.9 mm in controls vs. 63.2 ± 6.5 mm in HFrEF; LA diameter: 36.5 ± 3.2 mm vs. 46.5 ± 5.8 mm; both *p* < 0.001; P-trend < 0.001). The proportion of patients with advanced symptoms (NYHA class III–IV) rose in parallel with HF severity, from 29.4% in HFpEF to 50.0% in HFmrEF and 71.4% in HFrEF (*p* < 0.001; P-trend < 0.001). Among biochemical parameters, troponin I concentrations were significantly higher in HF patients compared with controls, with the highest values observed in HFrEF (*p* < 0.001; P-trend < 0.001). LDL cholesterol and triglyceride levels differed significantly across groups and showed significant linear trends (P-trend = 0.02 and 0.03, respectively), whereas HDL cholesterol did not differ significantly between groups (*p* = 0.21). HbA1c showed a non-significant trend toward higher values across HF phenotypes (*p* = 0.09; P-trend = 0.06).

### 3.3. Serum NT-proBNP Levels Across Study Groups

**Figure 1:**
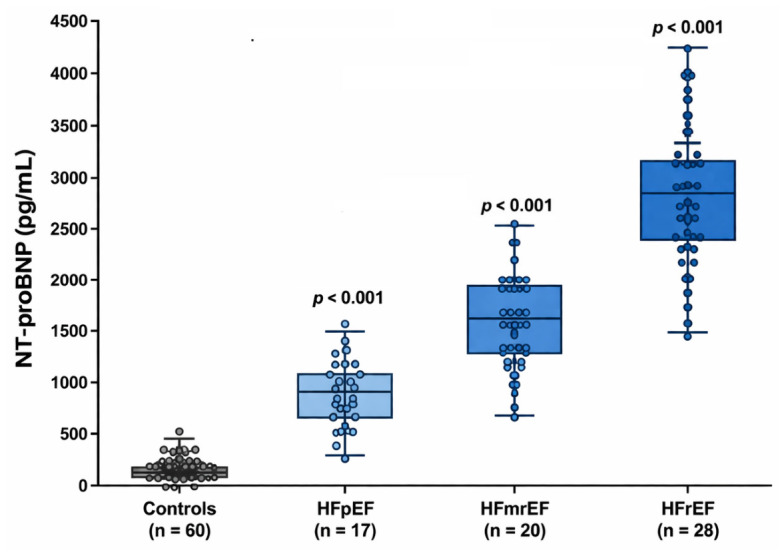
Box-and-whisker plots illustrate the distribution of serum NT-proBNP levels across control subjects and HF phenotypes.

Serum NT-proBNP concentrations differed markedly across study groups (Table 3). Overall, NT-proBNP was substantially elevated in HF patients compared with controls (mean ± SD: 1845 ± 620 vs. 95.7 ± 40.5 pg/mL). A clear stepwise gradient was observed across HF phenotypes, with mean levels rising from HFpEF (920.9 ± 310.3 pg/mL) to HFmrEF (1620.8 ± 480.2 pg/mL) and reaching the highest values in HFrEF (2850.6 ± 710.4 pg/mL). One-way ANOVA confirmed a highly significant overall difference across groups (F[3, 121] = 86.4, *p* < 0.001, partial η^2^ = 0.68). All three HF groups had significantly higher NT-proBNP than controls (all *p* < 0.001), with large pairwise effect sizes (Cohen’s d: 1.32, 1.86, and 2.45 for HFpEF, HFmrEF, and HFrEF, respectively). Post hoc comparisons further confirmed significant differences between all HF phenotypes (HFpEF vs. HFmrEF: *p* = 0.01; HFmrEF vs. HFrEF: *p* = 0.003; HFpEF vs. HFrEF: *p* < 0.001). The distribution of NT-proBNP levels across groups is illustrated in Figure 1, which highlights the progressive increase in median values and the greater dispersion observed with advancing HF severity. Few outliers were present, and their exclusion did not materially alter the study findings.

**Figure 2:**
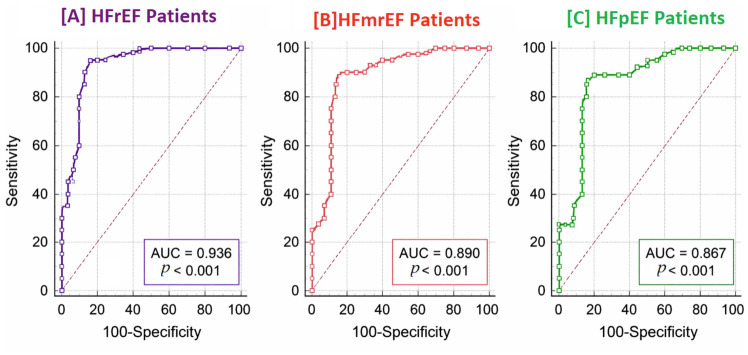
Receiver operating characteristic (ROC) curves for serum NT-proBNP levels across HF phenotypes. (**A**) HFrEF patients; (**B**) HFmrEF patients; (**C**) HFpEF patients vs. controls.

Receiver operating characteristic analysis (Figure 2) confirmed the diagnostic performance of NT-proBNP across HF phenotypes. The area under the ROC curve was 0.867 (95% CI: 0.78–0.93) for HFpEF, 0.890 (95% CI: 0.82–0.95) for HFmrEF, and 0.936 (95% CI: 0.88–0.97) for HFrEF (all *p* < 0.001), demonstrating phenotype-graded discriminatory accuracy with the highest performance observed in HFrEF.

### 3.4. Association Between NT-proBNP and Cardiac Function, Remodeling, and Clinical Severity

**Figure 3:**
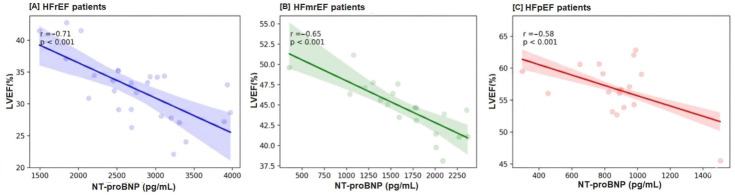
Scatter plots with fitted linear regression lines for the association between serum levels of NT-proBNP (pg/mL) and LVEF (%) in patients with (**A**) HFrEF (*n* = 28), (**B**) HFmrEF (*n* = 20), and (**C**) HFpEF (*n* = 17).

Correlations between NT-proBNP and clinical, biochemical, and echocardiographic parameters are detailed in Table 4. NT-proBNP exhibited a strong inverse correlation with LVEF across all groups, with the magnitude increasing from controls (r = −0.42, 95% CI: −0.61 to −0.18) through HFpEF (r = −0.58, 95% CI: −0.84 to −0.14) and HFmrEF (r = −0.65, 95% CI: −0.86 to −0.29) to HFrEF (r = −0.71, 95% CI: −0.86 to −0.44; all *p* < 0.001). Figure 3 displays scatter plots with fitted regression lines for each HF phenotype, visually confirming the progressive strengthening of the NT-proBNP–LVEF relationship with worsening systolic dysfunction.

Positive correlations were observed between NT-proBNP and markers of cardiac remodeling across all HF phenotypes. The associations with LVEDD and LA diameter were strongest in HFrEF (r = 0.66 and r = 0.61, respectively) and progressively weaker, though still significant, in HFmrEF (r = 0.59 and r = 0.55) and HFpEF (r = 0.52 and r = 0.49; all *p* < 0.001). NYHA functional class (III–IV) was positively correlated with NT-proBNP in all HF groups, with the strongest association in HFrEF (r = 0.68), followed by HFmrEF (r = 0.60) and HFpEF (r = 0.54; all *p* < 0.001). Troponin I showed moderate positive correlations with NT-proBNP that followed a similar phenotype-dependent gradient.

Metabolic parameters showed modest associations with NT-proBNP. Positive correlations were observed for LDL, triglycerides, and HbA1c, while HDL showed an inverse correlation (*p* < 0.05 for all). However, these correlations were substantially weaker than those observed for cardiac parameters, and several confidence intervals included or approached zero, indicating less robust and less consistent relationships.

### 3.5. Multivariate Regression Analysis of Determinants of NT-proBNP

Multivariable linear regression analysis was performed to identify independent determinants of log-transformed NT-proBNP levels (Table 5). The model included age, BMI, LVEF, LVEDD, LA diameter, NYHA class, troponin I, LDL, HDL, triglycerides, and HbA1c as independent variables, and was significant overall (F = 28.9, *p* < 0.001), explaining 68% of the variance in NT-proBNP (adjusted R^2^ = 0.68). Among all variables examined, LVEF was the strongest independent predictor, as indicated by the largest standardized coefficient (β = −0.42, *p* < 0.001). LVEDD (β = 0.33, *p* < 0.001), NYHA functional class (β = 0.36, *p* < 0.001), and LA diameter (β = 0.21, *p* = 0.003) were also independently associated with NT-proBNP. Troponin I demonstrated a modest independent association (β = 0.19, *p* = 0.003). Among metabolic parameters, only triglycerides (β = 0.12, *p* = 0.047) and HbA1c (β = 0.13, *p* = 0.039) retained independent associations, while LDL and HDL did not (*p* = 0.10 and *p* = 0.21, respectively). Age and BMI were not independently associated with NT-proBNP after adjustment for cardiac parameters. All variance inflation factors were below 5, indicating acceptable multicollinearity.

## 4. Discussion

The present study demonstrates that serum NT-proBNP concentrations exhibit a clear, stepwise gradient across the spectrum of heart failure, rising progressively from HFpEF to HFmrEF to HFrEF, and that this gradient is accompanied by consistent, phenotype-dependent associations with systolic dysfunction, adverse structural remodeling, and clinical symptom burden. These findings, derived from a single, well-characterized cohort integrating biochemical, echocardiographic, and clinical parameters, provide additional evidence that NT-proBNP reflects multiple dimensions of HF severity, including ventricular dysfunction, structural remodeling, and symptom burden, beyond its established role as a marker of hemodynamic stress. Nevertheless, the interpretation of NT-proBNP should remain individualized, as circulating concentrations are influenced by several patient-related factors beyond heart failure severity alone. Variables such as age, renal function, obesity, atrial fibrillation, and treatment status may substantially modify NT-proBNP levels and should be considered when interpreting NT-proBNP values in clinical practice and when designing future biomarker-based risk stratification strategies.

The phenotype-dependent gradient observed in this study is biologically coherent and aligns with the distinct pathophysiological drivers operating across the HF spectrum. In HFrEF, the markedly elevated NT-proBNP levels reflect the combined effects of pronounced ventricular dilation, increased wall stress, and sustained neurohormonal activation, all of which potentiate natriuretic peptide secretion from the failing myocardium [10,21]. In contrast, the lower concentrations observed in HFpEF are consistent with a pathophysiology in which elevated filling pressures and diastolic dysfunction, rather than overt systolic impairment and volume overload, constitute the primary hemodynamic stimulus for NT-proBNP release [21]. Importantly, NT-proBNP levels in HFpEF remained significantly elevated compared with controls, confirming that even in the absence of reduced LVEF, myocardial wall stress is sufficiently increased to drive meaningful biomarker elevation. The intermediate position of HFmrEF, both in NT-proBNP concentrations and in most echocardiographic indices, reinforces the concept that this phenotype represents a transitional state along a continuous disease spectrum rather than a discrete biological entity [22]. Our findings are also consistent with recent evidence highlighting the clinical significance of natriuretic peptides beyond diagnostic applications. A recent systematic review and meta-analysis by Ammar et al. demonstrated the prognostic utility of BNP and NT-proBNP in patients with HFpEF, emphasizing their value for risk stratification and adverse outcome prediction. Although our study was not designed to evaluate prognosis, our observation that NT-proBNP exhibits phenotype-specific gradients and significant associations with structural remodeling and symptom burden further supports the concept that natriuretic peptides capture multiple dimensions of heart failure pathophysiology and should be interpreted within an integrated phenotype-oriented framework [23].

A central finding of this study is the demonstration that NT-proBNP maintains significant associations with both functional and structural indices across all HF phenotypes, albeit with varying strength. The inverse correlation with LVEF and the positive correlations with LVEDD and LA diameter were present in HFpEF, HFmrEF, and HFrEF, indicating that NT-proBNP captures the shared axis of adverse remodeling that underlies HF progression irrespective of LVEF category. The graded strengthening of these correlations from HFpEF to HFrEF is instructive: it suggests that the coupling between natriuretic peptide release and cardiac structure becomes tighter as ventricular dilation and systolic dysfunction become more pronounced. These observations should also be interpreted in the context of cardiac remodeling and reverse remodeling processes, which have become central therapeutic targets in contemporary heart failure management. Effective implementation of guideline-directed medical therapy (GDMT) may attenuate ventricular dilation, improve systolic performance, reduce myocardial wall stress, and consequently lower circulating natriuretic peptide concentrations [24]. Conversely, persistent adverse remodeling may contribute to sustained elevations in NT-proBNP and progressive deterioration of cardiac function. Therefore, the relationship between NT-proBNP and LVEF should be viewed as a dynamic interaction that may evolve over time in response to therapeutic interventions, rather than as a static association [25]. This observation is consistent with prior work by Iwanaga et al. [26], who reported stronger BNP remodeling correlations in dilated cardiomyopathy compared with diastolic HF, and extends those findings to NT-proBNP in a fully stratified cohort. The weaker correlations observed in HFpEF, while still statistically significant, likely reflect the greater heterogeneity of this phenotype, the lesser contribution of volumetric remodeling, and the influence of comorbidities, particularly obesity. The relationship between obesity and NT-proBNP is complex and is often described as the obesity–natriuretic peptide paradox, whereby obese individuals may exhibit disproportionately lower circulating natriuretic peptide concentrations despite significant cardiac dysfunction. In addition, obesity may independently affect cardiac structure and function through increased plasma volume, altered ventricular loading conditions, systemic inflammation, and metabolic dysregulation. These factors may attenuate the association between NT-proBNP and conventional echocardiographic indices, particularly in HFpEF, and underscore the need for individualized interpretation of biomarker levels in obese patients [27,28].

The strong association between NT-proBNP and NYHA functional class across all phenotypes warrants particular attention. This finding suggests that NT-proBNP is associated not only with the structural and hemodynamic derangements of HF but also with their functional expression as experienced by the patient [29]. The observed relationship between objective cardiac abnormalities and symptom severity further supports the potential clinical utility of NT-proBNP beyond diagnosis alone. However, given the cross-sectional design, the role of NT-proBNP in gauging disease activity or monitoring therapeutic response remains speculative and should be evaluated in prospective longitudinal studies [30]. The phenotype graded strength of this association, strongest in HFrEF and weakest in HFpEF, parallels the pattern observed for structural indices and suggests that in HFpEF, factors beyond cardiac remodeling, including peripheral mechanisms and comorbid conditions, may contribute more substantially to symptom generation [31].

The ROC analysis further substantiates the phenotype-dependent discriminatory performance of NT-proBNP. The AUC gradient was 0.867 for HFpEF, 0.890 for HFmrEF, and 0.936 for HFrEF, which mirrors the stepwise increase in mean NT-proBNP concentrations and the progressive separation from controls. The excellent discriminatory accuracy in HFrEF is expected and consistent with the substantial body of evidence supporting NT-proBNP as a diagnostic tool in this phenotype [32]. The lower AUC in HFpEF, while still representing good discriminatory performance, reflects the inherently more challenging diagnostic context: HFpEF patients often have NT-proBNP levels that overlap with those of controls, particularly in the presence of obesity or when assessed during compensated states [33]. This finding underscores the importance of interpreting NT-proBNP in conjunction with echocardiographic and clinical data in suspected HFpEF, rather than relying on biomarker thresholds in isolation.

Integration of emerging biomarkers of heart failure destabilization provides a more comprehensive framework for interpreting the clinical significance of NT-proBNP in the present study. Beyond natriuretic peptides, a growing body of evidence supports the use of multi-biomarker strategies incorporating markers of myocardial injury, systemic inflammation, and multiorgan congestion to improve risk stratification and early identification of impending decompensation. In this context, biomarkers such as high-sensitivity cardiac troponins, soluble suppression of tumorigenicity-2 (sST2), and growth differentiation factor-15 (GDF-15) have demonstrated incremental prognostic value when used alongside natriuretic peptides, particularly in patients with chronic and acutely decompensated HF. Recent evidence further suggests that integrated biomarker panels may outperform single-marker approaches in capturing the complex pathophysiological transitions underlying HF destabilization, including subclinical myocardial injury and evolving haemodynamic congestion [34,35]. Beyond conventional circulating biomarkers, emerging multimodal approaches that integrate NT-proBNP with complementary physiological and biochemical parameters, including arterial blood gas and electrolyte assessments, may further enhance diagnostic precision and support individualized management strategies across the spectrum of heart failure. Future studies should investigate the incremental value of these integrated approaches in phenotype-specific heart failure assessment [36].

Taken together, these findings generate several hypotheses that may inform future clinical practice and research. First, they suggest that NT-proBNP may contribute to future phenotype-based risk stratification approaches that move beyond LVEF alone to incorporate biomarkers reflecting multiple disease dimensions. Such an approach may be particularly valuable in HFmrEF and HFpEF, although this hypothesis requires validation in prospective studies before clinical implementation. Second, the consistent associations between NT-proBNP and remodeling indices raise the possibility that serial NT-proBNP measurements could serve as a non-invasive surrogate for tracking adverse remodeling or its reversal during therapy, a hypothesis that requires prospective testing in longitudinal studies with paired biomarker and imaging assessments [37]. Third, the phenotype-specific differences in correlation strength suggest that the same NT-proBNP concentration may carry different biological meaning depending on the underlying HF phenotype; this observation argues against uniform biomarker thresholds and favors phenotype-informed interpretation.

This study has several methodological strengths, including the inclusion of all major HF phenotypes within a single cohort, the use of age- and sex-matched controls, standardized echocardiographic and biochemical measurements, and comprehensive statistical analyses incorporating effect sizes and multivariable adjustment. However, several limitations must be acknowledged. The cross-sectional design precludes assessment of longitudinal biomarker trajectories and their relationship to disease progression or therapeutic response. In addition, the relatively modest sample size, particularly within the individual HF phenotype subgroups, may have limited statistical power for subgroup analyses and reduced our ability to comprehensively account for all potential confounding variables. Although age and BMI were included in the multivariable model, other clinically relevant determinants of NT-proBNP concentrations, including atrial fibrillation and renal dysfunction, were not systematically collected and therefore could not be incorporated into the analyses [12,38]. Consequently, residual confounding cannot be excluded, and the findings should be interpreted with appropriate caution. NT-proBNP was measured at a single time point, and biological variability over time was not captured [37]. In addition, detailed information regarding guideline-directed medical therapy, including treatment duration, dose optimization, and therapeutic intensity, was not systematically collected. Since GDMT may substantially influence cardiac remodeling, reverse remodeling, and natriuretic peptide concentrations, its potential confounding effect could not be assessed in the present study [24,25]. The sample sizes within individual phenotype subgroups, particularly HFpEF (*n* = 17), may limit the precision of subgroup-specific correlation estimates and the stability of ROC-derived metrics; the wider confidence intervals observed for HFpEF correlations reflect this constraint. Renal function was assessed only for eligibility purposes at the severe impairment threshold (eGFR < 30 mL/min/1.73 m^2^), and detailed renal function parameters were not systematically collected for analytical adjustment. Likewise, information regarding atrial fibrillation status was unavailable. Since both renal dysfunction and atrial fibrillation are established determinants of natriuretic peptide concentrations, their absence from the analyses may have contributed to residual confounding [39]. All participants were recruited from a single tertiary cardiac center, which may limit generalizability to community-based or primary care populations. Finally, the absence of an external validation cohort means that the reported AUC values and correlation coefficients require independent confirmation before translation into clinical practice. In addition, the relatively small number of patients within individual HF phenotypes, particularly the HFpEF subgroup, may have limited the statistical power of subgroup analyses and affected the stability of phenotype-specific ROC-derived threshold estimates. Therefore, these findings should be interpreted as exploratory and hypothesis-generating, pending validation in larger and more diverse populations. Future research should address these limitations through multicenter, prospective, longitudinal study designs incorporating serial biomarker and imaging assessments, comprehensive renal function characterization, and external validation of diagnostic and prognostic thresholds. Investigation of whether phenotype-specific NT-proBNP cutoffs improve diagnostic accuracy or prognostic discrimination over uniform thresholds would be of clinical value.

## 5. Conclusions

Herein, serum NT-proBNP concentrations demonstrated a clear phenotype-dependent gradient rising progressively from HFpEF through HFmrEF to HFrEF and exhibited robust, consistent associations with left ventricular systolic dysfunction, adverse structural remodeling, and clinical symptom severity. These associations were present across all phenotypes, with a graded increase in strength paralleling the severity of systolic impairment and ventricular dilation. These findings further support the concept that NT-proBNP may function as an integrative biomarker in heart failure, as its concentrations were associated with functional impairment, structural remodeling, and clinical symptom burden across different HF phenotypes within a single study population. The observation that NT-proBNP remained significantly associated with indices of cardiac remodeling and symptom burden, even in HFpEF, where diagnostic uncertainty is often greatest, underscores its potential clinical relevance. The phenotype-dependent gradient of NT-proBNP and the differing strengths of its associations across HF categories are consistent with the concept that biomarker behavior may vary across the spectrum of HF phenotypes. However, given the cross-sectional design, these findings should be interpreted as associative rather than causal and do not permit conclusions regarding disease progression, reverse remodeling, treatment response monitoring, or risk stratification. Instead, these observations generate hypotheses that warrant confirmation in future prospective longitudinal studies specifically designed to evaluate temporal changes, therapeutic response, and clinical outcomes. Nevertheless, the relatively small sample size, particularly in the HFpEF subgroup, warrants cautious interpretation, and these findings should be validated in larger multicenter studies.

## Figures and Tables

**Table 1:** General characteristics of study participants stratified by HF phenotypes and controls.

Variable	Controls (*n* = 60)	HFrEF (*n* = 28)	HFmrEF (*n* = 20)	HFpEF (*n* = 17)	*p*-Value *
**Age (years)**	57.2 ± 9.8	59.1 ± 10.6	58.4 ± 9.9	57.8 ± 10.1	0.82
**Gender**					
**Male (%)**	38 (63.3%)	19 (67.9%)	13 (65.0%)	10 (58.8%)	0.79
**Female (%)**	22 (36.7%)	9 (32.1%)	7 (35.0%)	7 (41.2%)	0.76
**BMI (kg/m^2^)**	28.6 ± 4.1	29.5 ± 4.5	28.9 ± 4.2	28.7 ± 4.0	0.73
**Diabetes Mellitus (%)**	31 (51.7%)	15 (53.6%)	10 (50.0%)	9 (52.9%)	0.96
**Hypertension (%)**	29 (48.3%)	14 (50.0%)	10 (50.0%)	8 (47.1%)	0.99
**Smoking (%)**	15 (25.0%)	7 (25.0%)	5 (25.0%)	4 (23.5%)	0.99

**BMI**, body mass index; **HFrEF**, heart failure with reduced ejection fraction; **HFmrEF**, heart failure with mildly reduced ejection fraction; **HFpEF**, heart failure with preserved ejection fraction. Data are expressed as mean ± SD or *n* (%). * Group comparisons were performed using one-way ANOVA with Tukey’s post hoc test for continuous variables and chi-square test for categorical variables. A *p*-value < 0.05 was considered statistically significant.

**Table 2:** Clinical, biochemical, and echocardiographic characteristics across HF phenotypes and controls.

Variable	Controls (*n* = 60)	HFrEF (*n* = 28)	HFmrEF (*n* = 20)	HFpEF (*n* = 17)	*p*-Value *	P-Trend **
**LVEF (%)**	61.5 ± 3.8	32.4 ± 5.1	44.6 ± 3.2	56.8 ± 4.1	<0.001	<0.001
**LVEDD (mm)**	48.2 ± 3.9	63.2 ± 6.5	56.4 ± 5.2	50.8 ± 4.6	<0.001	<0.001
**LA Diameter (mm)**	36.5 ± 3.2	46.5 ± 5.8	42.1 ± 4.7	39.3 ± 3.9	<0.001	<0.001
**NYHA Class (III–IV, %)**	0 (0%)	20 (71.4%)	10 (50.0%)	5 (29.4%)	<0.001	<0.001
**Troponin I (ng/mL)**	0.0	0.18 ± 0.06	0.12 ± 0.04	0.08 ± 0.03	<0.001	<0.001
**LDL (mmol/L)**	1.80 ± 0.60	2.95 ± 0.72	2.71 ± 0.65	2.58 ± 0.58	<0.05	0.02
**HDL (mmol/L)**	1.32 ± 0.34	1.05 ± 0.28	1.10 ± 0.30	1.14 ± 0.27	0.21	0.08
**Triglycerides (mmol/L)**	1.45 ± 0.52	1.98 ± 0.64	1.82 ± 0.58	1.78 ± 0.55	<0.05	0.03
**HbA1c (%)**	5.6 ± 0.7	7.2 ± 1.1	6.9 ± 1.0	6.7 ± 0.9	0.09	0.06

Data are expressed as mean ± SD for continuous variables and *n* (%) for categorical variables. **HbA1c:** glycated hemoglobin; **LVEF**, left ventricular ejection fraction; **LVEDD**, left ventricular end-diastolic diameter; **LA**, left atrial; **NYHA**, New York Heart Association; **LDL**, low-density lipoprotein; **HDL**, high-density lipoprotein; **HFrEF**, heart failure with reduced ejection fraction; **HFmrEF**, heart failure with mildly reduced ejection fraction; **HFpEF**, heart failure with preserved ejection fraction. * Group comparisons were performed using one-way ANOVA with Tukey’s post hoc test for continuous variables and chi-square test for categorical variables. ** P-trend was calculated using linear trend analysis across increasing heart failure severity (controls → HFpEF → HFmrEF → HFrEF.

**Table 3:** Phenotype-dependent differences in serum NT-proBNP concentrations across HF.

Group	*n*	NT-proBNP(pg/mL) Mean ± SD *	*p*-Value vs. Controls	Pairwise Post Hoc *p*-Value			Cohen’s d		
				HFpEF	HFmrEF	HFrEF	HFpEF	HFmrEF	HFrEF
**Controls**	60	95.7 ± 40.5	—	—	—	—	—	—	—
**HFpEF**	17	920.9 ± 310.3	<0.001	—	0.01	<0.001	—	1.32	2.45
**HFmrEF**	20	1620.8 ± 480.2	<0.001	0.01	—	0.003	1.32	—	1.86
**HFrEF**	28	2850.6 ± 710.4	<0.001	<0.001	0.003	—	2.45	1.86	—

* Data are presented as mean ± standard deviation (SD). Group comparisons were performed using one-way ANOVA followed by Tukey’s post hoc multiple comparison test. Overall ANOVA: F(3, 121) = 86.4, *p* < 0.001, partial η^2^ = 0.68. Between-group effect size is expressed as partial eta squared (η^2^); pairwise effect sizes are expressed as Cohen’s d. **HFpEF**: Heart Failure with preserved Ejection Fraction; **HFmrEF**: Heart Failure with mildly reduced Ejection Fraction; **HFrEF**: Heart Failure with reduced Ejection Fraction.

**Table 4:** Correlations between NT-proBNP and clinical, biochemical, and echocardiographic parameters across HF phenotypes.

Variable	Controls		HFrEF		HFmrEF		HFpEF		*p*-Value
	r *	95% CI	r *	95% CI	r *	95% CI	r *	95% CI	
**LVEF (%)**	−0.42	−0.61–−0.18	−0.71	−0.86–−0.44	−0.65	−0.86–−0.29	−0.58	−0.84–−0.14	<0.001
**LVEDD (mm)**	0.38	0.14–0.58	0.66	0.35–0.84	0.59	0.19–0.82	0.52	0.06–0.80	<0.001
**LA Diameter (mm)**	0.35	0.10–0.55	0.61	0.28–0.81	0.55	0.13–0.80	0.49	0.02–0.78	<0.001
**NYHA Class (III–IV)**	—	—	0.68	0.38–0.85	0.60	0.21–0.82	0.54	0.08–0.81	<0.001
**Troponin I (ng/mL)**	—	—	0.53	0.18–0.76	0.47	0.04–0.75	0.42	−0.06–0.74	<0.001
**LDL (mmol/L)**	0.20	−0.06–0.43	0.34	−0.04–0.64	0.30	−0.16–0.65	0.27	−0.22–0.65	0.04
**HDL (mmol/L)**	−0.18	−0.42–0.08	−0.31	−0.62–0.07	−0.28	−0.63–0.18	−0.25	−0.63–0.24	0.04
**Triglycerides (mmol/L)**	0.22	−0.04–0.45	0.38	0.01–0.67	0.33	−0.12–0.67	0.29	−0.20–0.67	0.03
**HbA1c (%)**	0.24	−0.02–0.47	0.40	0.04–0.68	0.36	−0.09–0.69	0.32	−0.17–0.69	0.02

* Data are presented as Pearson correlation coefficients (r) with corresponding 95% confidence intervals (CI). Correlation analyses were performed using Pearson’s correlation test. A two-tailed *p*-value < 0.05 was considered statistically significant. **LVEF**, left ventricular ejection fraction; **LVEDD**, left ventricular end-diastolic diameter; **LA**, left atrial; **NYHA**, New York Heart Association; **LDL**, low-density lipoprotein; **HDL**, high-density lipoprotein; **HbA1c**, glycated hemoglobin; **HFrEF**, heart failure with reduced ejection fraction; **HFmrEF**, heart failure with mildly reduced ejection fraction; **HFpEF**, heart failure with preserved ejection fraction.

**Table 5:** Multivariate linear regression analysis for independent determinants of log-transformed NT-proBNP levels.

Variable	β Coefficient	Standard Error	Standardized β	95% CI	*p*-Value
**Age (years)**	0.012	0.006	0.14	0.001–0.023	0.032
**BMI (kg/m^2^)**	−0.018	0.009	−0.12	−0.035–−0.001	0.038
**LVEF (%)**	−0.045	0.007	−0.42	−0.059–−0.031	<0.001
**LVEDD (mm)**	0.031	0.006	0.33	0.019–0.043	<0.001
**LA Diameter (mm)**	0.024	0.008	0.21	0.008–0.040	0.003
**NYHA Class (III–IV)**	0.52	0.11	0.36	0.30–0.74	<0.001
**Troponin I (ng/mL)**	0.88	0.29	0.19	0.31–1.45	0.003
**LDL (mmol/L)**	0.067	0.041	0.10	−0.014–0.148	0.10
**HDL (mmol/L)**	−0.091	0.073	−0.08	−0.235–0.053	0.21
**Triglycerides (mmol/L)**	0.072	0.036	0.12	0.001–0.143	0.047
**HbA1c (%)**	0.058	0.028	0.13	0.003–0.113	0.039

Data are presented as β coefficients (unstandardized), standard error (SE), standardized β, and 95% confidence intervals (CI). NT-proBNP was log-transformed prior to analysis. Multicollinearity was assessed using variance inflation factor (VIF < 5). Statistical significance was set at *p* < 0.05. Model fit: R^2^ = 0.71; adjusted R^2^ = 0.68; F = 28.9; *p* < 0.001.

## Data Availability

Data sharing is applicable to this article.

## References

[1] Shahim B., Kapelios C.J., Savarese G., Lund L.H. (2023). Global Public Health Burden of Heart Failure: An Updated Review. Card. Fail. Rev..

[2] Fonseca C., Baptista R., Franco F., Moura B., Pimenta J., Moraes Sarmento P., Cardoso J.S., Brito D. (2025). Worsening Heart Failure: Progress, Pitfalls, and Perspectives. Heart Fail. Rev..

[3] Schwinger R.H.G. (2021). Pathophysiology of Heart Failure. Cardiovasc. Diagn. Ther..

[4] Lüscher T.F. (2021). Classification of Heart Failure: A Farewell to Ejection Fraction?. Anatol. J. Cardiol..

[5] McDonagh T.A., Metra M., Adamo M., Gardner R.S., Baumbach A., Böhm M., Burri H., Butler J., Čelutkienė J., Chioncel O. (2023). 2023 Focused Update of the 2021 ESC Guidelines for the Diagnosis and Treatment of Acute and Chronic Heart Failure: Developed by the Task Force for the Diagnosis and Treatment of Acute and Chronic Heart Failure of the European Society of Cardiology (ESC) with the special contribution of the Heart Failure Association (HFA) of the ESC. Eur. Heart J..

[6] Kim T., Sheen M., Ryan D., Joseph J. (2026). Addressing Unmet Needs in Heart Failure with Preserved Ejection Fraction: Multi-Omics Approaches to Therapeutic Discovery. Int. J. Mol. Sci..

[7] Giangregorio F., Centenara E., Mazzocchi S., Gerra L., Tursi F., Imberti D., Aschieri D. (2025). Assessing Venous Congestion in Acute and Chronic Heart Failure: A Review of Splanchnic, Cardiac and Pulmonary Ultrasound: Part 1: Conventional B-Mode, Colordoppler, and Vexus Protocol. J. Clin. Med..

[8] Panagopoulou V., Deftereos S., Kossyvakis C., Raisakis K., Giannopoulos G., Bouras G., Pyrgakis V., Cleman M.W. (2013). NTproBNP: An Important Biomarker in Cardiac Diseases. Curr. Top. Med. Chem..

[9] Kanyal S., Das A., Bashir A.M.D., Syed A.H., Aujla S., Chaudhary J., Patel D., Goel A., Kanyal S., Das A. (2025). N-Terminal Pro-B-Type Natriuretic Peptide (NT-ProBNP) as a Biomarker in Heart Failure with Preserved Ejection Fraction (HFpEF) Versus Heart Failure with Reduced Ejection Fraction (HFrEF): The Way Forward in the Age of Proteomics. Cureus.

[10] Lainscak M., von Haehling S., Anker S.D. (2009). Natriuretic Peptides and Other Biomarkers in Chronic Heart Failure: From BNP, NT-ProBNP, and MR-ProANP to Routine Biochemical Markers. Int. J. Cardiol..

[11] Madamanchi C., Alhosaini H., Sumida A., Runge M.S. (2014). Obesity and Natriuretic Peptides, BNP and NT-ProBNP: Mechanisms and Diagnostic Implications for Heart Failure. Int. J. Cardiol..

[12] Takase H., Dohi Y. (2014). Kidney Function Crucially Affects B-Type Natriuretic Peptide (BNP), N-Terminal ProBNP and Their Relationship. Eur. J. Clin. Investig..

[13] Mouzarou A., Hadjigeorgiou N., Melanarkiti D., Plakomyti T.E. (2025). The Role of NT-ProBNP Levels in the Diagnosis of Hypertensive Heart Disease. Diagnostics.

[14] Birrell H., Isles C., Fersia O., Anwar M., Mondoa C., McFadyen A. (2024). Assessment of the Diagnostic Value of NT-ProBNP in Heart Failure with Preserved Ejection Fraction. Br. J. Cardiol..

[15] Chrysohoou C., Konstantinou K., Tsioufis K. (2024). The Role of NT-ProBNP Levels in the Diagnosis and Treatment of Heart Failure with Preserved Ejection Fraction—It Is Not Always a Hide-and-Seek Game. J. Cardiovasc. Dev. Dis..

[16] Creegan D., Starling R.C., Hameed A., Daly M.J., O’Neill J. (2026). The Diagnosis and Subclassification of Heart Failure with Preserved Ejection Fraction: A Review. Heart Fail. Rev..

[17] Cao Z., Jia Y., Zhu B. (2019). BNP and NT-ProBNP as Diagnostic Biomarkers for Cardiac Dysfunction in Both Clinical and Forensic Medicine. Int. J. Mol. Sci..

[18] Fatima A., Mansoor M.H., Munir A., Ali B., Maryam H., Tariq M.S. (2025). Evaluation of N-Terminal Pro-B-Type Natriuretic Peptide (NT-ProBNP) as a Biomarker of Cardiac Dysfunction Across Heart Failure Phenotypes. Cureus.

[19] Bielecka-Dabrowa A., Gluba-Brzózka A., Michalska-Kasiczak M., Misztal M., Rysz J., Banach M. (2015). The Multi-Biomarker Approach for Heart Failure in Patients with Hypertension. Int. J. Mol. Sci..

[20] Lang R.M., Badano L.P., Mor-Avi V., Afilalo J., Armstrong A., Ernande L., Flachskampf F.A., Foster E., Goldstein S.A., Kuznetsova T. (2015). Recommendations for Cardiac Chamber Quantification by Echocardiography in Adults: An Update from the American Society of Echocardiography and the European Association of Cardiovascular Imaging. Eur. Heart J. Cardiovasc. Imaging.

[21] Reddy Y.N.V., Borlaug B.A. (2016). Heart Failure with Preserved Ejection Fraction. Curr. Probl. Cardiol..

[22] Meijers W.C., van der Velde A.R., de Boer R.A. (2016). Biomarkers in Heart Failure with Preserved Ejection Fraction. Neth. Heart J..

[23] Ammar L.A., Massoud G.P., Chidiac C., Booz G.W., Altara R., Zouein F.A. (2024). BNP and NT-ProBNP as Prognostic Biomarkers for the Prediction of Adverse Outcomes in HFpEF Patients: A Systematic Review and Meta-Analysis. Heart Fail. Rev..

[24] Furquim S.R., de Sousa Lira M.T.S., de Sá Pereira Belfort D., Biselli B., Chizzola P.R., Munhoz R.T., Bocchi E.A., Ayub-Ferreira S.M. (2025). Reverse Remodelling, Myocardial Recovery and Remission in Heart Failure with Reduced Ejection Fraction: Clinical Implications and Management Strategies. Card. Fail. Rev..

[25] Myhre P.L., Vaduganathan M., Claggett B.L., Miao Z.M., Jhund P.S., de Boer R.A., Hernandez A.F., Inzucchi S.E., Kosiborod M.N., Lam C.S.P. (2022). Influence of NT-ProBNP on Efficacy of Dapagliflozin in Heart Failure with Mildly Reduced or Preserved Ejection Fraction. JACC Heart Fail..

[26] Iwanaga Y., Nishi I., Furuichi S., Noguchi T., Sase K., Kihara Y., Goto Y., Nonogi H. (2006). B-Type Natriuretic Peptide Strongly Reflects Diastolic Wall Stress in Patients with Chronic Heart Failure: Comparison Between Systolic and Diastolic Heart Failure. J. Am. Coll. Cardiol..

[27] Iwanaga Y., Kihara Y., Niizuma S., Noguchi T., Nonogi H., Kita T., Goto Y. (2007). BNP in Overweight and Obese Patients with Heart Failure: An Analysis Based on the BNP-LV Diastolic Wall Stress Relationship. J. Card. Fail..

[28] Peng L., Zeng T., Quan E., Pan S., Li B., Wen Z., Xiong Z., Zhao Y. (2025). Obesity Phenotypes Causally Affect Cardiac MRI Structure and Induced Non-Ischaemic Cardiomyopathy. Card. Fail. Rev..

[29] Song B.G., Jeon E.S., Kim Y.H., Kang M.K., Doh J.H., Kim P.H., Ahn S.J., Oh H.L., Kim H.J., Sung J.D. (2005). Correlation Between Levels of N-Terminal Pro-B-Type Natriuretic Peptide and Degrees of Heart Failure. Korean J. Intern. Med..

[30] Sado G., Kemp Gudmundsdottir K., Bonander C., Ekström M., Engdahl J., Svennberg E. (2024). The Role of NT-ProBNP in Screening for Atrial Fibrillation in Hypertensive Disease. IJC Heart Vasc..

[31] Shah S.J., Katz D.H., Deo R.C. (2014). Phenotypic Spectrum of Heart Failure with Preserved Ejection Fraction. Heart Fail. Clin..

[32] Pintea A.M., Minciună I.A., Pop D. (2026). Biomarkers for Screening and Diagnosis of Heart Failure in Cardiovascular–Kidney–Metabolic Syndrome: A Narrative Review. Int. J. Mol. Sci..

[33] van Dalen B.M., Chin J.F., Motiram P.A., Hendrix A., Emans M.E., Brugts J.J., Westenbrink B.D., de Boer R.A. (2025). Challenges in the Diagnosis of Heart Failure with Preserved Ejection Fraction in Individuals with Obesity. Cardiovasc. Diabetol..

[34] Parlati A.L.M., Madaudo C., Nuzzi V., Manca P., Gentile P., Di Lisi D., Jordán-Ríos A., Shamsi A., Manzoni M., Sadler M. (2025). Biomarkers for Congestion in Heart Failure: State-of-the-Art and Future Directions. Card. Fail. Rev..

[35] Berger M., März W., Niessner A., Delgado G., Kleber M., Scharnagl H., Marx N., Schuett K. (2024). IL-6 and HsCRP Predict Cardiovascular Mortality in Patients with Heart Failure with Preserved Ejection Fraction. ESC Heart Fail..

[36] Tetaj N., Segreti A., Piccirillo F., Pelullo M., Crispino S.P., Ciancio M., Ussia G.P., Grigioni F., Tetaj N., Segreti A. (2026). Diagnostic and Prognostic Value of Arterial Blood Gas and Electrolyte Analyses in Heart Failure. Rev. Cardiovasc. Med..

[37] Rusconi P.G., Ludwig D.A., Ratnasamy C., Mas R., Harmon W.G., Colan S.D., Lipshultz S.E. (2010). Serial Measurements of Serum NT-ProBNP as Markers of Left Ventricular Systolic Function and Remodeling in Children with Heart Failure. Am. Heart J..

[38] Bularga A., Hung J., Daghem M., Stewart S., Taggart C., Wereski R., Singh T., Meah M.N., Fujisawa T., Ferry A.V. (2022). Coronary Artery and Cardiac Disease in Patients with Type 2 Myocardial Infarction: A Prospective Cohort Study. Circulation.

[39] Knudsen C.W., Omland T., Clopton P., Westheim A., Wu A.H.B., Duc P., McCord J., Nowak R.M., Hollander J.E., Storrow A.B. (2005). Impact of Atrial Fibrillation on the Diagnostic Performance of B-Type Natriuretic Peptide Concentration in Dyspneic Patients: An Analysis from the Breathing Not Properly Multinational Study. J. Am. Coll. Cardiol..

